# IMPLICON: an ultra-deep sequencing method to uncover DNA methylation at imprinted regions

**DOI:** 10.1093/nar/gkaa567

**Published:** 2020-07-04

**Authors:** Tajda Klobučar, Elisa Kreibich, Felix Krueger, Maria Arez, Duarte Pólvora-Brandão, Ferdinand von Meyenn, Simão Teixeira da Rocha, Melanie Eckersley-Maslin

**Affiliations:** Instituto de Medicina Molecular, João Lobo Antunes, Faculdade de Medicina, Universidade de Lisboa, 1649-028 Lisboa, Portugal; Epigenetics Programme, Babraham Institute, Cambridge CB22 3AT, UK; Bioinformatics Group, Babraham Institute, Cambridge CB22 3AT, UK; Instituto de Medicina Molecular, João Lobo Antunes, Faculdade de Medicina, Universidade de Lisboa, 1649-028 Lisboa, Portugal; Instituto de Medicina Molecular, João Lobo Antunes, Faculdade de Medicina, Universidade de Lisboa, 1649-028 Lisboa, Portugal; Epigenetics Programme, Babraham Institute, Cambridge CB22 3AT, UK; Instituto de Medicina Molecular, João Lobo Antunes, Faculdade de Medicina, Universidade de Lisboa, 1649-028 Lisboa, Portugal; Epigenetics Programme, Babraham Institute, Cambridge CB22 3AT, UK

## Abstract

Genomic imprinting is an epigenetic phenomenon leading to parental allele-specific expression. Dosage of imprinted genes is crucial for normal development and its dysregulation accounts for several human disorders. This unusual expression pattern is mostly dictated by differences in DNA methylation between parental alleles at specific regulatory elements known as imprinting control regions (ICRs). Although several approaches can be used for methylation inspection, we lack an easy and cost-effective method to simultaneously measure DNA methylation at multiple imprinted regions. Here, we present IMPLICON, a high-throughput method measuring DNA methylation levels at imprinted regions with base-pair resolution and over 1000-fold coverage. We adapted amplicon bisulfite-sequencing protocols to design IMPLICON for ICRs in adult tissues of inbred mice, validating it in hybrid mice from reciprocal crosses for which we could discriminate methylation profiles in the two parental alleles. Lastly, we developed a human version of IMPLICON and detected imprinting errors in embryonic and induced pluripotent stem cells. We also provide rules and guidelines to adapt this method for investigating the DNA methylation landscape of any set of genomic regions. In summary, IMPLICON is a rapid, cost-effective and scalable method, which could become the gold standard in both imprinting research and diagnostics.

## INTRODUCTION

Genomic imprinting describes the parent-of-origin dependent monoallelic expression of ∼100–200 genes in mammals (reviewed in (1)). The inherited set of maternal and paternal chromosomes are not equivalent and are both required for full-term development (2,3). This effect maps to specific chromosomal regions (4), which later were discovered to contain imprinted genes (5–8). Imprinted genes are important regulators of foetal growth and development (reviewed in (9,10)) and involved in several postnatal endocrine and metabolic pathways, as well as in neuronal functions affecting behaviour and cognition (reviewed in (1)). Not surprisingly, genetic or epigenetic disturbances resulting in altered dosage of imprinted genes lead to severe developmental, neurological and metabolic diseases in humans (reviewed in (11,12)), such as the Prader-Willi (PWS) (OMIM#176270) and Angelman (AS) (OMIM#105830) syndromes caused by defects in the paternal or maternal chr15q11-q13 region, respectively (reviewed in (13,14)).

Most imprinted genes are located in clusters throughout the genome, containing a *cis*-acting CpG-rich DNA element referred to as imprinting control region (ICR) (reviewed in (1)). The ICR is epigenetically marked by DNA methylation in a parent-of-origin fashion, which correlates with expression and/or silencing of the surrounding imprinted genes. Deletions of ICRs result in the loss of parental allele-specific expression within an imprinted cluster (15,16). ICRs acquire parental-allele specific DNA methylation in the germline, which are maintained throughout development and adulthood, resisting the global wave of demethylation and *de novo* methylation steps during early embryonic development (reviewed in (1,17,18)). The preservation of parental allele-specific methylation at ICRs, also known as germline differentially methylated regions (gDMRs), is fundamental for the correct maintenance of imprinted expression throughout life.

Despite their importance, there is currently no robust, cost-effective and high-throughput method to assess the methylation status of ICRs across multiple imprinted regions (Supplementary Table S1; reviewed in (19)). DNA methylation is commonly assessed using methods based on bisulfite sequencing (20). Treatment of DNA with sodium bisulfite results in deamination of unmodified cytosines to uracils, whereas methylated cytosines remain unchanged. Traditionally, this is followed by PCR amplification of a region of interest followed by subcloning and Sanger sequencing—a laborious and costly process when considering multiple samples or viewpoints. Alternatively, bisulfite-treated DNA can be converted into a next-generation sequencing library to give genome-wide information, which is expensive when at least 10-fold genomic coverage is required to accurately determine methylation levels at individual ICRs (21,22). This can be somewhat mitigated using reduced representation bisulfite sequencing (RRBS) to enrich for CpG-rich regions of the genome including imprinted regions (23,24), or array-based methods, as the Illumina Infinium methylation BeadChip (25). These methods, however, take several weeks, require advanced analysis skills and are not feasible to be routinely performed at scale. At the other end, pyrosequencing analysis of bisulfite converted DNA provides an easier method to analyse a few (<5) CpGs within ICRs, but is not high-throughput (26). Bisulfite-free approaches have also been used to study genomic imprinting such as genome-wide Methylated DNA Immunoprecipitation sequencing (MeDIP-seq) (27,28) and Enzymatic Methyl-seq (EM-seq) (29). An advantage of EM-seq or other similar bisulfite-free methods such as Tet-assisted pyridine borane (TAPS-seq) (30) is that they can be coupled with long read sequencing (EM-LR-seq and lrTAPS) (29,31) overcoming the limitation of read length unavoidable in bisulfite methods that lead to DNA fragmentation. Long read sequencing itself using Oxford Nanopore can directly detect methylated Cs to measure parental allele-specific methylation on long stretches of DNA (32), however this or analogous technology such as PacBio Single-Molecule Real-Time (SMRT) are still hindered by limited coverage or throughput and high costs (33).

Importantly, in recent years, researchers have adapted the traditional bisulfite-PCR method amplification involving subcloning and Sanger sequencing to have a next-generation sequencing output (34). This targeted deep bisulfite sequencing approach addresses many of the shortfalls of other methods, enabling regions of interest to be probed in a fast, cost-effective and high-resolution manner. We reasoned that adapting this amplicon method to assess genomic imprints will provide an invaluable and much needed tool for the experimental and clinical science communities.

Here, we present IMPLICON (IMPrint ampLICON), an ultra-deep sequencing method to robustly measure DNA methylation levels with base-pair resolution at imprinted regions. This method uses bisulfite-treated DNA to generate amplicon sequencing libraries covering most murine and human imprinted regions. This way, IMPLICON generates base-resolution datasets with over 1000-fold coverage that can be quickly and easily analysed to determine genomic imprinting fidelity in <6 days. Furthermore, we provide detailed instructions to analyse the data, and rules for designing additional primer sequences, making this method easily adaptable to analyse DNA methylation patterns at other genomic regions of interest. We expect that this rapid, scalable and cost-effective ultra-deep sequencing method will become a powerful tool for both imprinting research and diagnostics.

## MATERIALS AND METHODS

### Biological material

Inbred mouse genomic DNA samples were obtained from the Babraham Institute C57BL/6 (BL6) J/Babr Ageing Mouse Colony as previously described (35). Genomic DNA samples from F1 hybrid animals were obtained from BL6 × CAST/EiJ (CAST) reciprocal crosses from the iMM JLA Rodent Facility. Animals were housed in a maximum of four per cage in a temperature-controlled room (24°C) with a 12-h light/dark cycle. Animals were fed standard CRM (P) VP diet and both food diet and water were available *ad libitum*. All experiments involving mice were carried out in accordance with the UK and Portugal Government Home Office licensing procedures.

Genomic DNA from human peripheral blood was collected from two healthy female volunteers via fingerprick. Human embryonic stem cells (ESC) genomic material was collected from H9-KN2 (*Nanog-Klf2*) ESCs (36) and cultured in six-well dishes under naïve (N2B27 supplemented with human LIF, 1 mM Chir, 1 mM PD03 and 2 mM Go6983 on MEF feeder cells) and primed (Vitronectin in E8 media) conditions as previously described (37). Genomic material was also obtained from human primary fibroblasts (AS Fib. and Ctrl Fib.) and respective induced pluripotent stem cell (iPSC) (Ctrl D, Ctrl E, AS A, AS B, AS D and AS E) lines from an Angelman patient and sex- and age-matched healthy individual as previously described (38). Briefly, primary fibroblasts were maintained in DMEM supplemented with 10% fetal bovine serum, 1 mM l-glutamine and 100 units/ml penicillin, 100 μg/ml streptomycin (Life Technologies). iPSCs were cultured in mTeSR1 medium (STEMCELL Technologies) supplemented with 50 units/ml penicillin, 50 μg/ml streptomycin (Life Technologies) in Matrigel (Corning)-coated plates. All cell lines grew in a humid incubator at 37°C with 5% (v/v) CO_2_.

### DNA extraction and bisulfite treatment

Genomic DNA was isolated using either conventional phenol:chloroform:isoamyl alcohol extraction, the DNeasy Blood and Tissue Kit (Qiagen) or the AllPrep DNA/RNA Micro Kit (Qiagen) according to manufacturer's instructions and eluted into TE buffer or H_2_O. 1μg of genomic DNA was bisulfite converted using the EZ DNA methylation Gold kit (Zymo Research) according to manufacturer's instructions with either magnetic bead or column clean-up and eluted in 66 μl elution buffer to obtain a final concentration of ∼15 ng/μl bisulfite-converted DNA.

### IMPLICON primer design and testing

Genomic coordinates for murine ICRs or other differentially methylated regions of interest (gDMRs or somatic differentially methylated regions – sDMRs) were obtained from the former webpage, https://atlas.genetics.kcl.ac.uk, and validated using in-house DNA methylation datasets. Appropriate SNPs in the vicinity of ICRs were acquired either from the literature (39) or from https://www.sanger.ac.uk/sanger/Mouse_SnpViewer/rel-1505. Human imprinting genomic coordinates for gDMRs were defined using oocyte and sperm methylomes (40). Genomic DNA sequences of the regions of interest were obtained from UCSC Genome Browser (https://www.genome.ucsc.edu) and imported into MethPrimer (https://www.urogene.org/methprimer/) (41) or BiSearch (http://bisearch.enzim.hu/) (42). For each region, at least two primer pairs for bisulfite sequencing PCR were designed, selecting those with smaller product size (optimal size 300 bp, max 430 bp), a minimum of five CpGs in the PCR product, and no CpGs within the PCR primers. The following sequence was added to the forward (CTACACGACGCTCTTCCGATCT) and reverse (TGCTGAACCGCTCTTCCGATCTNNNNNNNN) primers (where N denotes a random nucleotide to generate a unique molecular identifier -UMI). Each primer pair PCR condition combination was tested on 2 ng bisulfite-treated genomic DNA, 0.3 μM forward and reverse primer and 2× KAPA HiFi Uracil+ ReadyMix with the following conditions: initial denaturation at 95°C for 5 min, 30–35 cycles of 98°C denaturation for 20 s, variable annealing temperature for 15 s and extension at 72°C for 60 s; followed by a final extension at 72°C for 10 min. The annealing temperature was tested between 60 and 72°C. PCR products were run on a 1% agarose–TAE gel and those yielding a single strong band were selected for inclusion in the amplicon assay. Approximately 50% of designed primers yield a single strong band under these conditions. The use of 2× KAPA HiFi Uracil+ ReadyMix is crucial to ensure efficient amplification despite the lower complexity of bisulfite-treated DNA.

### IMPLICON library preparation

The IMPLICON protocol consists of two PCR reactions. In the first reaction, each sample is amplified with each primer pair in individual reactions: 30 ng (2 μl of 66 μl eluted) of bisulfite-treated DNA) is amplified with 1.2 μl of a 10 μM primer pool (final 1.5 μM), containing both forward and reverse primers, and 4 μl of 2× KAPA HiFi Uracil+ ReadyMix in a final volume of 8 μl. The hybrid mouse samples were processed in a final volume of 16 μl. While lower starting amounts of bisulfite-treated DNA can be used, we do not recommend this as it may not accurately reflect DNA methylation levels of the original population and may not make use of the full sequencing depth of IMPLICON due to a higher duplication rate. DNA was amplified using the following conditions: initial denaturation at 95°C for 5 min, 30 cycles of 98°C denaturation for 20 s, variable annealing temperature for 15 s and extension at 72°C for 60 s; followed by a final extension at 72°C for 10 min. Annealing temperatures for each primer pair were optimized as described above and are listed in Supplementary Table S2. All PCR reactions for each individual sample were pooled together and cleaned-up using 1.5× AMPure XP beads and eluted in 20 μl H_2_O. In the second PCR reaction, barcoded Illumina adapters are attached to the pooled PCR samples ensuring that each sample pool receives a unique reverse barcoded adapter. The 20 μl PCR pool was amplified using 1 μl of 10 μM Illumina PE1.0 primer (same for all samples), 1 μl of 10 μM Illumina iTAG primer (distinct for each sample) and 25 μl 2× KAPA HiFi Uracil+ ReadyMix in a 50 μl reaction using the following conditions: initial denaturation at 98°C for 45 s, 5 cycles of 98°C denaturation for 15 s, 65°C annealing for 30 s and extension at 72°C for 30 s; followed by a final extension at 72°C for 5 min. Reactions were cleaned-up with 1x AMPure XP beads and eluted in 20 μl H_2_O. Libraries were verified by running 1:30 dilutions on an Agilent bioanalyzer. Note that the profile of these libraries is spikey due to their amplicon nature (Supplementary Figure S1C). Libraries were sequenced using the Illumina MiSeq platform to generate paired-end 250 bp reads using 10% PhIX spike-in as the libraries are of low complexity.

### IMPLICON sequencing analysis

Data was processed using standard Illumina base-calling pipelines. As the first step in the processing, the first 8 bp of Read 2 were removed and written into the readID of both reads as an in-line barcode or UMI. This UMI was then later used during the de-duplication step with ‘deduplicate bismark –barcode mapped_file.bam’. Raw sequence reads were then trimmed to remove both poor quality calls and adapters using Trim Galore (v0.6.2 for hybrid mouse tissues, v0.4.4 for human and inbred mouse tissues, www.bioinformatics.babraham.ac.uk/projects/trim_galore/, Cutadapt version 2.3 for hybrid mouse tissues, v1.9.1 for human and inbred mouse tissues, parameters: –paired). Trimmed reads were aligned to the mouse or human reference genome in paired-end mode. Alignments were carried out with Bismark v0.14.4 for hybrid mouse tissues and v0.18.2 for human and inbred mouse tissues (43). CpG methylation calls were extracted from the mapping output using the Bismark methylation extractor (v0.22.1 for hybrid mouse tissues, v0.18.2 for human and inbred mouse tissues). De-duplication was then carried out with deduplicate_bismark, using the –barcode option to take UMIs into account (see above). For hybrid mouse strain experiments, the data was aligned to a hybrid genome of BL6/CAST (the genome was prepared with the SNPsplit package (v0.3.4, https://github.com/FelixKrueger/SNPsplit). Following alignment and de-duplication, reads were split allele-specifically with SNPsplit. Aligned read (.bam) files were imported into Seqmonk software (http://www.bioinformatics.babraham.ac.uk/projects/seqmonk) for all downstream analysis. Probes were made for each CpG contained within the amplicon and quantified using the DNA methylation pipeline or total read count options. Downstream analysis was performed using Excel and GraphPad.

From the raw data deposited in GEO under the accession number GSE146129, the reads mapped to the following murine (mm10) and human (hg38) genomic coordinates were excluded for consideration in this article for one of the following reasons: (i) failure to reach the coverage threshold (>100 reads); (ii) clear sequencing bias towards the methylated or unmethylated amplicons or to one of the SNPs, (iii) regions out of the scope of this article. For inbred mice data: Chr1:63264732–63264796, Chr2:152686485–152686582, Chr2:174328905–174329102, Chr6:4746303–4746438, Chr6:58906821–58907146 and Chr17:12742173–12742420; for hybrid mice data: Chr6:4746303–4746438, Chr18:12973031–12973038 and Chr18:36988436–36988740; for human data: Chr19:16555181–16555319, Chr3:181712902–181713043 and Chr20:37521191–37521391.

For a detailed step-by-step guide of data processing analysis, see https://github.com/FelixKrueger/IMPLICON.

## RESULTS

### IMPLICON design

**Figure 1.**
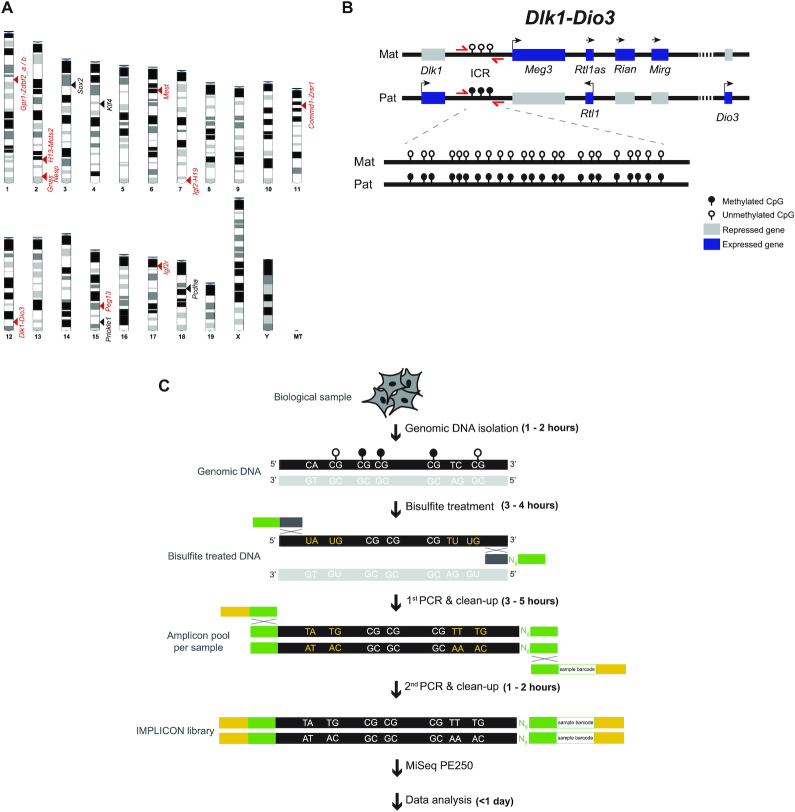
The IMPLICON method. (**A**) Schematic view of the murine karyotype depicting the location of the regions detected by IMPLICON; black arrowheads – control regions; red arrowheads – imprinted regions. (**B**) Schematic representation of the mouse *Dlk1-Dio3* imprinted cluster; Mat – Maternal inherited chromosome; Pat – Paternal inherited chromosome; ICR – imprinting control region; red arrows – primers to amplify *Dlk1-Dio3* ICR; genomic region is not drawn to scale. (**C**) Brief scheme of the IMPLICON method and its approximate timeline; bisulfite treatment of genomic DNA converts unmethylated cytosines to uracils (yellow letters), whilst methylated cytosines are retained as cytosines (white letters). Two rounds of PCR are then performed: the first PCR amplifies each region for each sample separately and adds eight random nucleotides (N_8_) for data de-duplication and adapter sequences; after pooling amplicons for each biological sample, a second PCR completes a sequence-ready library with sample-barcodes for multiplexing. White lollipops – unmethylated CpGs; black lollipops – methylated CpGs; black DNA strand – targeted strand for amplification; light grey DNA strand – strand not targeted for amplification; Dark grey and green boxes - primers annealing the targeted strand (dark grey), containing adapter sequences (green); green and yellow – primers annealing the adapters (green), containing barcoded Illumina adapters and Illumina PE1.0 primer sequence (yellow).

To surpass the current limitations for methylation analysis at imprinted regions, we adapted bisulfite-treated amplicon next-generation sequencing (NGS) approaches for these loci, naming our method IMPrint ampLICON or IMPLICON (Figure 1A–C). We designed primers targeting well characterized murine imprinted regions and added adapter sequences to allow for efficient library construction (see Materials and Methods). The following rules were used for primer design: (i) primer sequences do not contain CpGs to ensure both methylated and unmethylated alleles are amplified equally; (ii) the maximum size of amplified regions is ideally <300 bp and no >430 bp to reduce any bias introduced from bisulfite treatment-induced DNA fragmentation; (iii) amplified regions contain a minimum of five CpGs and (iv) primers yield a single PCR product when tested on bisulfite-treated genomic DNA (Supplementary Figure S1A, B; Table S2). We also designed control primers against regions consistently unmethylated (promoter and 5′end of *Sox2* and *Klf4* genes) and methylated (intronic CpG-rich region of the *Pcdha* gene cluster and last exon of the *Prickle1* gene) in mouse ESCs to control for bisulfite conversion efficiency (Supplementary Table S2). Importantly, and in contrast to previous amplicon bisulfite sequencing methods (34), primers also included a random 8 nucleotide barcode or UMI to enable post-sequencing data de-duplication. Primers were individually validated to give a single product on bisulfite-treated genomic DNA from mouse embryonic stem cells (ESCs) or tissue samples (Supplementary Figure S1A, B), resulting in 15 primer pairs covering nine murine imprinted clusters (Figure 1A; Supplementary Table S2).

The IMPLICON method consists of two PCR reactions. The first PCR is an individual reaction for each primer pair which are then pooled together by sample, followed by a second PCR using barcoded Illumina adapters for sample identification (Figure 1C, see Materials and Methods). Representative bioanalyzer traces after the first and second PCRs are displayed in Supplementary Figure S1C. The final set of 19 primer pairs generate PCR products sampling a total of 245 CpGs (range from 5 to 23, on average 13 CpGs per amplicon) (Supplementary Table S2). Up to 32 samples can be easily processed simultaneously to generate an amplicon library in just 2–3 days, which is subsequently sequenced on an Illumina MiSeq platform (Figure 1C).

### IMPLICON in inbred mice

As a proof of principle, we analysed DNA methylation levels in mouse organs (heart, liver and lung) from three independent adult male C57BL/6J mice of different ages (3 months, 6 months and 15 months) for which imprints are well known to be maintained (50%:50% methylated/unmethylated ratio) (22,44,45). With just one MiSeq run, we were able to examine each CpG within the selected genomic regions with an average of ∼4900-fold coverage after post-sequencing de-duplication. The UMI in the PCR primer enabled us to control for PCR amplification bias which ranged from a minimum of 0.6% to a maximum of 72.1% of duplicated reads (median of 18.2%) (Supplementary Table S3). Importantly, this enabled us to determine if methylated and unmethylated reads were amplified to the same extent. With our selected primers, we saw very little, if any, changes in overall DNA methylation levels before and after de-duplication (Supplementary Figure S2). Furthermore, the de-duplication step guarantees that each original DNA molecule is measured only once in the final dataset, ensuring the ultra-deep dataset has single-molecule resolution.

**Figure 2.**
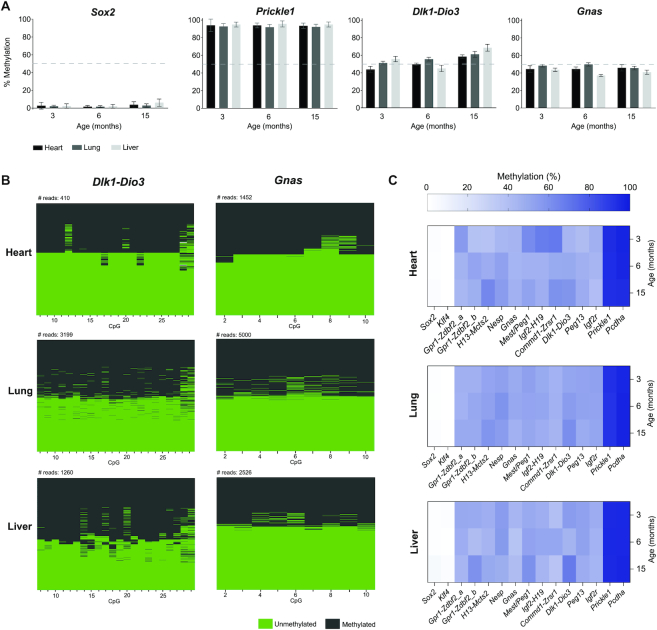
IMPLICON in adult tissues from C57BL/6J mice. (**A**) Methylation analysis of *Sox2* (unmethylated control), *Prickle1* (methylated control), and the *Dlk1-Dio3* and *Gnas* ICRs in heart, lung and liver tissues from adult mice of different ages: 3 months, 6 months and 15 months; Graph represents the mean ± SD methylation levels measured at each CpG within each genomic region for each individual mouse; dashed line marks 50% level of methylation. (**B**) Plot displaying methylated and unmethylated CpGs for each CpG position (in columns) in all the individual reads (in rows) for both the *Dlk1-Dio3* and *Gnas* (for the *Gnas*_SNP primer) imprinted loci in the heart, lung and liver of a 3-month-old mouse. (**C**) Heatmap displaying average methylation levels at individual imprinted regions and controls (in columns) from adult mice of different ages (in rows) in heart, lung and liver.

As predicted, both unmethylated (*Sox2* and *Klf4*) and methylated controls (*Pcdha* and *Prickle1*) showed, respectively, low (<∼10%) or high (>∼90%) levels of DNA methylation for all tested tissues in the three individuals (Figure 2A; Supplementary Table S3). In contrast, we observed DNA methylation levels of ∼50% for *Dlk1-Dio3* and *Gnas* imprinted regions that did not change as a function of the organ or age (Figure 2A). Examining DNA methylation consistency for each read confirmed that the 50% methylation levels reflected an equal mix of unmethylated (<∼10%) and methylated (>∼90%) reads as expected for imprinted regions (Figure 2B). DNA methylation levels of ∼50% were also seen for all other imprinted regions analysed by IMPLICON (Figure 2C, Supplementary Table S3).

### IMPLICON in hybrid mice from reciprocal crosses

**Figure 3.**
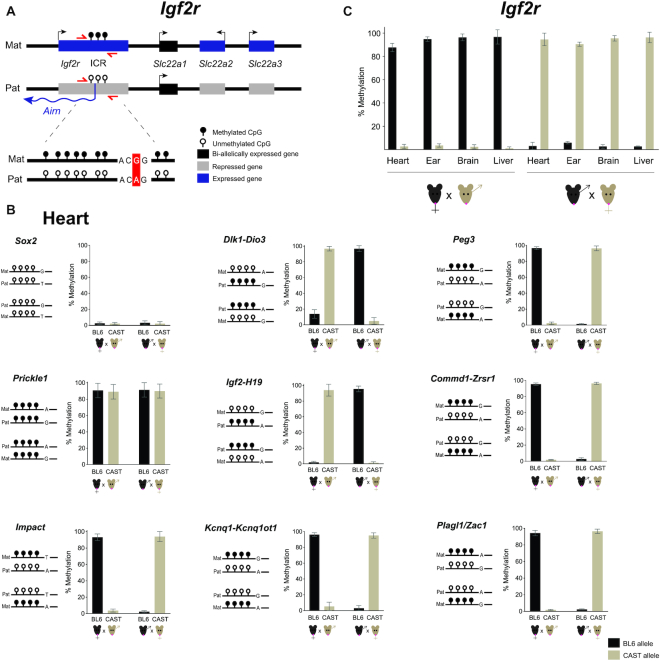
Allele-specific IMPLICON in F1 adult tissues from C57BL/6J x Cast/EiJ reciprocal crosses. (**A**) Schematic representation of the *Igf2r* imprinted cluster; Mat – Maternally inherited chromosome; Pat – Paternally inherited chromosome; ICR – imprinting control region; red arrows – primers to amplify *Igf2r* ICR; in the scheme below, a single nucleotide polymorphism is highlighted in red; genomic region not drawn to scale. (**B**) Methylation analysis of *Sox2* (unmethylated control), *Prickle1* (methylated control) and ICRs of imprinted regions in the heart from F1 hybrid adult mice derived from C57BL/6J × CAST/EiJ reciprocal crosses; Graph represents the mean ± SD methylation levels measured at each CpG within different genomic regions per parental allele in the two F1 hybrid mice; Scheme on the left of each graph represents the expected methylation status of each region (white lollipops – unmethylated CpGs; black lollipops – methylated CpGs; Mat – maternal allele; Pat – paternal allele; black mice (BL6) – C56BL/6J strain; brown mice (CAST) – CAST/EiJ strain; regions are not drawn to scale. (**C**). Methylation analysis for the *Igf2r* imprinted cluster in heart, ear, brain and liver of F1 hybrid mice from reciprocal crosses; Graph represents the mean ± SD methylation levels measured for all the CpGs within the *Igf2r* ICR in each parental allele per mouse.

To validate the parent-of-origin methylation differences, single nucleotide polymorphisms (SNPs) of hybrid mouse crosses were used to differentiate between maternal and paternal reads. We generated reciprocal crosses of two distinct mouse strains, BL6 and CAST, which are widely used for allele-specific studies owing to the frequent presence of SNPs (22,46) (Figure 3A). From the original set of primers used in inbred mice, only six contained appropriate SNPs within the amplified region. Thus, we redesigned 10 more primer pairs according to the rules above to include SNPs not masked by bisulfite conversion (C/T SNPs were excluded) within the region of interest, covering 13 imprinted regions, 2 unmethylated and 1 methylated control regions (see Materials and Methods; Supplementary Figure S3; Table S2)

We tested this allele-specific version of IMPLICON in different organs (heart, liver, brain and ear) from two F1 hybrid BL6/CAST adult male mice from reciprocal crosses (BL6 female × CAST male and vice-versa). We generated IMPLICON libraries with an average allelic coverage across the sampled CpGs that reached as high as 20 000-fold. Importantly, roughly the same proportion of reads were assigned to both the BL6 (51.26%) and CAST (48.54%) genomes, arguing against amplification bias with only 0.20% of reads left unassigned (Supplementary Table S3).

For the unmethylated (*Sox2* and *Klf4*) and methylated (*Prickle1*) controls, our results show, respectively, low (<∼10%) or high (>∼90%) methylation levels in both maternal and paternal hybrid alleles in the heart, but also the other tissues (Figure 3B; Supplementary Table S3). Confident in the control regions, we turned our attention to ICRs. As exemplified in Figure 3B, at the *Dlk1-Dio3* and *Igf2-H19* (paternally methylated) and *Peg3, Commd1-Zrsr1, Impact, Kcnq1-Kcnq1ot1* and *Plagl1/Zac1* (maternally methylated) ICRs in the heart the maternal allele was always unmethylated or methylated, respectively, independent of the strain-specific SNP. These parental allele-specific methylation patterns were unequivocally shown for all imprinted regions analysed in the four tissue samples of the same mouse (Supplementary Table S3) as exemplified for the maternally methylated *Igf2r* locus (Figure 3C). In summary, we were able to adapt the IMPLICON method for the screening of methylation at multiple ICRs with allelic discrimination.

### Human IMPLICON

After our success in implementing IMPLICON for mouse imprinted regions, we created a human version of IMPLICON. Published methylome data from human oocytes and sperm (40) were analysed to accurately determine the genomic coordinates of gDMRs. As controls for bisulfite conversion, we included amplicons targeting regions fully unmethylated (promoter and TSS of *KLF4*) or methylated (last exon of *RHOG*) in primed human ESCs (hESCs) (Supplementary Figure S4; Table S2). Unfortunately, for some regions, such as the *DLK1-DIO3* locus, we were unable to design specific primers according to our rules, which can be due to high repeat or CpG density in these regions. Nonetheless, we were able to capture 14 human imprinted clusters (12 oocyte gDMRs and 2 sperm gDMRs) using 16 primer pairs, in addition to the control regions.

**Figure 4.**
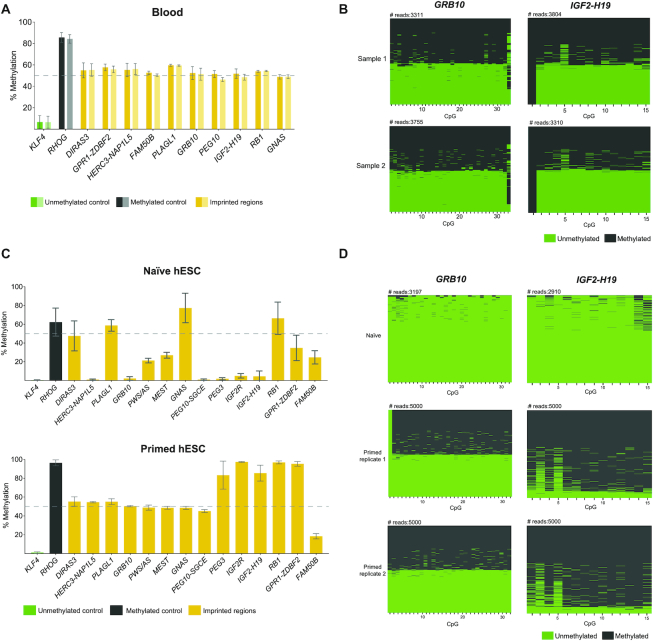
Human IMPLICON in blood, naïve and primed hESCs. (**A**) Methylation analysis of the *KLF4* (unmethylated control), *RHOG* (methylated control) and several gDMRs in blood samples from two independent individuals; Graph represents the mean ± SD methylation levels measured at each CpG within each genomic region for each individual blood sample (sample 1: dark colours; sample 2: light colours); dashed line marks 50% level of methylation. (**B**) Plot displaying methylated and unmethylated CpGs for each CpG position (in columns) in all the individual reads (in rows) for *GRB10* and *IGF2-H19* for each individual blood sample (Sample 1 and 2). (**C**) Methylation analysis of the unmethylated (*KLF4*), methylated (*RHOG*) controls and several gDMRs in naïve and primed hESCs; Graph represents the mean ± SD methylation levels measured at each CpG within each genomic region; primed hESCs shown here are an average of two replicates, whereas for naïve hESCs only one sample was analysed; dashed line marks 50% level of methylation. (**D**) Plot displaying methylated and unmethylated CpGs for each CpG position (in columns) in all the individual reads (in rows) for *GRB10* and *IGF2-H19* for individual hESC samples (one naïve and two primed hESC replicates).

We first tested these primers in blood samples from two healthy individuals. Once again, we obtained high coverage for the CpGs analysed (average of ∼6500-fold). Our unmethylated and methylated controls showed generally low levels of DNA methylation for *KLF4* (<∼10%) and high levels of methylation for *RHOG* (>∼90%) (Figure 4A). As expected, all the gDMRs inspected showed methylation levels of ∼50% (Figure 4A) reflecting an equal mix of methylated (>∼90%) and unmethylated (>∼10%) reads in accordance with normal imprinting patterns (Figure 4B). Overall, these results suggest that our IMPLICON approach is suitable to look at multiple human imprinted regions.

Then, we used IMPLICON to assess imprinting fidelity in different hESC culture conditions. Human ESCs cultured in naïve conditions have globally lower levels of DNA methylation (∼30% compared to 70–80% in conventional or primed hESC cultures). As a result, loss of imprinting methylation is frequently observed in naïve conditions, whilst primed hESCs are more able to maintain imprinting fidelity (47–49). In our IMPLICON results, the unmethylated control region (*KLF4*) showed <∼10% methylation as anticipated (Figure 4C). Reflecting the expected global levels of DNA methylation, the *RHOG* methylated control region showed higher (>∼90%) levels of DNA methylation in primed hESCs, in comparison to naïve hESCs (∼50%) (Figure 4C). Of the 14 imprinted regions analysed, 8 showed the expected 50% DNA methylation levels in primed hESC cultures, whereas only three imprinted loci (*DIRAS3*, *PLAGL1* and *RB1*) maintained DNA methylation in naïve hESCs (Figure 4C). Naïve hESCs tend to lose DNA methylation, with 10 imprinted regions having less than the 40–60% expected DNA methylation levels, compared to just one locus, *FAM50B*, losing methylation in primed hESCs (Figure 4C). This was reflected appropriately in the number of fully methylated and unmethylated reads: at the *GRB10* locus primed hESCs presented the same proportion of these reads in two biological replicates, while only fully unmethylated reads were seen for naïve hESCs (Figure 4D). In primed conditions, five regions had close to 100% methylation (e.g. *IGF2-H19*), with only one hypermethylated region (*GNAS*) seen in naïve hESCs (Figure 4C). *IGF2-H19* is a perfect example of a region fully methylated in primed conditions and completely unmethylated in naïve conditions (Figure 4D). In summary, our analyses show that IMPLICON can be used successfully to identify imprinting errors in hESC cultures and furthermore highlights the importance of checking imprinting fidelity in hESC lines, including those in primed conditions.

**Figure 5.**
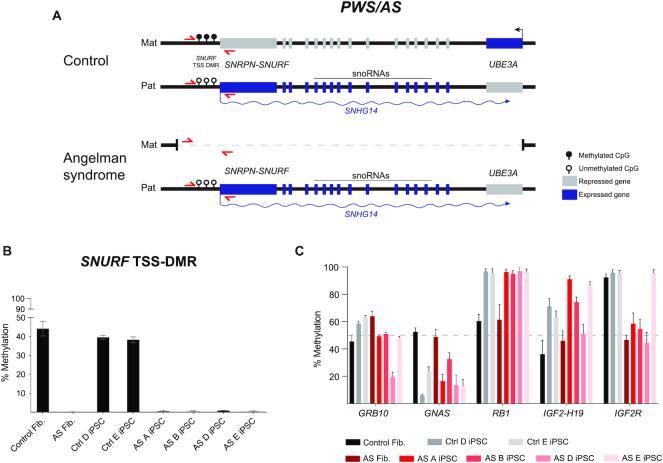
Human IMPLICON in donor fibroblasts and hiPSCs from an Angelman patient and healthy donor. (**A**) Schematic representation of the PWS/AS locus on chr15q11–13 in human neurons, in control (healthy donor) and Angelman patient (harbouring a 4.8 Mb deletion of the maternal PWS/AS locus); *SNURF* TSS-DMR is the ICR of this region; red arrows – primers to amplify *SNURF* TSS-DMR; genomic region not drawn to scale. (**B**) Methylation analysis of the *SNURF* TSS-DMR in donor fibroblasts and hiPSCs from an Angelman patient and healthy donor; Graph represents the mean ± SD methylation levels measured at each CpG within the *SNURF* TSS-DMR for each sample. (**C**) Methylation analysis of several imprinted regions (*GRB10*, *GNAS*, *RB1*, *IGF2-H19* and *IGF2R*) in donor fibroblasts and hiPSCs from an Angelman patient and healthy donor; Graph represents the mean ± SD methylation levels measured at each CpG within the *SNURF* TSS-DMR for each sample.

Next, we ran our human IMPLICON on dermal fibroblasts and corresponding human induced pluripotent stem cell (hiPSC) lines previously generated from an Angelman patient and a healthy individual (38) (Figure 5A) to search for putative imprinting defects often found in hiPSCs (50–52). As predicted, our unmethylated and methylated controls showed low (<∼10%) and high levels (>∼90%) of DNA methylation, respectively (Supplementary Table S3). We then screened for the *SNURF* TSS-DMR at the PWS/AS cluster which is only methylated on the maternally inherited allele and is deleted in the Angelman patient-derived cells (Figure 5A). While the healthy fibroblasts and hiPSCs showed the expected ∼50% methylation levels, the Angelman-derived cells presented ∼0% methylation, consistent with the absence of the methylated maternal *SNURF* TSS-DMR region (Figure 5A and B). For the other imprinted regions sampled, we found values around the expected ∼50% methylation in healthy and Angelman fibroblasts (Figure 5C; Supplementary Table S3). A remarkable exception was the *IGF2R* int2-DMR, the gDMR at the *IGF2R* imprinted locus, presenting ∼50% methylation in Angelman patient fibroblasts (and iPSCs), but >∼90% methylation in the healthy fibroblasts (and correspondent iPSCs). Interestingly, imprinting at this region is known to be polymorphic and to differ from individual to individual (53).

In contrast to stable imprinting associated with somatic cells, we observed many methylation aberrations at gDMRs for many of the imprinted clusters in hiPSCs (Figure 5C; Supplementary Table S3). In addition to hESCs, imprinted defects have been broadly associated with hiPSCs (50–52) and are one of the major concerns for the downstream applications of these cells (54,55). A few of the imprinted loci showed no or minor abnormalities (e.g. *DIRAS3*, *GPR1-ZDBF2, MEST*, *PEG10-SGCE, GRB10*), while others show consistent hypomethylation (e.g. *GNAS*) or hypermethylation (e.g. *RB1* and *PEG3*). Furthermore, several loci displayed considerable variation from iPSC-to-iPSC line (e.g. *NAP1L5*, *FAM50B*, *PLAGL1* and *IGF2-H19*) (Figure 4C; Supplementary Table S3). In comparison to previous reports on imprinted defects in hiPSCs that used methylome techniques (51,52), we obtained overlapping results showing a consistent tendency for hypomethylation (e.g. *PLAGL1* and *GNAS*), hypermethylation (e.g. *PEG3*), as well as, normal maintenance of imprinting (e.g. *MEST* and *PEG10*) (Supplementary Table S4). The *HERC3-NAP1L5* locus shows divergent results, however, no consistent results were observed for this locus among the previous reports (Supplementary Table S4). Overall, our IMPLICON technique identified methylation defects in gDMRs of hiPSCs consistent with previous reports highlighting its potential application in identifying imprinted defects in the context of human imprinted regions.

## DISCUSSION

We present IMPLICON to examine DNA methylation patterns at imprinted regions with an unprecedented coverage. We designed a set of primers to study both murine and human imprinted clusters, the former with allelic resolution. This method surpasses many shortcomings, such as time and cost of other methodologies to look at parental allele-specific methylation (Supplementary Table S1). We believe IMPLICON will provide an added value to the imprinting community and could become the gold standard for methylation inspection at multiple imprinted regions for both research and diagnostics.

IMPLICON is an adaptation of amplicon-bisulfite sequencing method assessing multiple imprinted regions and handling several samples in a single MiSeq run. Consequently, it outperforms commonly used DNA methylation analysis methods in many ways (Supplementary Table S1). Firstly, it is much less laborious and more high-throughput than bisulfite-PCR Sanger sequencing and pyrosequencing methods traditionally used to look at methylation in imprinted regions. IMPLICON yields considerably richer datasets with over 100-fold increment in the coverage of amplicons analysed (compared to bisulfite-PCR Sanger sequencing) over a longer stretch of CpGs (compared to pyrosequencing). Furthermore, the costs compared to whole genome bisulfite sequencing, RRBS and array-based methods makes our approach much more appealing. Moreover, the high coverage (>1000-fold) at imprinted regions of our approach with nucleotide and allelic resolution is not matched by any of these bisulfite-based methods. Finally, given the reduced costs and ultra-deep genomic coverage, IMPLICON could be easily scalable to include more genomic regions of interest and more samples.

In recent years, new technological advances enabled the emergence of bisulfite-free methods for DNA methylation analysis such as EM-seq or TAPS-seq (29–31) which, unlike bisulfite-based methods, are capable of distinguishing 5′methyl-cytosine (5mC) from 5′hydroxymethyl-cytosine (5hmC). However, as there are no parental-allele specific differences in 5hmC at ICRs (29) our simpler and cheaper bisulfite-based amplicon method will accurately detect imprinting aberrations. Another advantage of these methods is the reduced DNA fragmentation allowing for long-read sequencing (29,31). However, library preparation and sequencing costs are still prohibitive for a multiplex and scalable approach such as IMPLICON. In conclusion, we believe our IMPLICON method is the most cost-effective, scalable, rapid and ultra-deep approach that will best serve the epigenetic community at analysing methylation at multiple imprinted regions.

The mouse remains the favorite animal model for studying the underlying mechanisms of imprinting regulation (reviewed in (1)). For example, the use of mouse models allowed the identification of ZFP57 and ZFP445 KRAB zinc-finger proteins as fundamental protection factors of methylation imprints at critical developmental stages (56,57). Therefore, inspection of methylation remains highly relevant for murine imprinted regions. Our initial set of primers designed on the murine reference genome (C57BL/6) consisted of 15 primers covering 9 imprinted regions. Of note, we have previously used a subset of these primers to report loss of methylation at imprinted regions in 2C-like (MERVL^+^Zscan4^+^) mouse ESCs (58), attesting for the utility of our method. The additional primers designed for allele-specific IMPLICON are also suitable to analyse imprinted loci in C57BL/6 inbred mice, which increased the number of primers to 25 covering a total of 15 imprinted clusters. This set of IMPLICON primers could, therefore, be used to look at imprinting maintenance in particular circumstances (e.g. environmental insult or genetic ablation) in cells or animals of the C57BL/6 background. Most of the primer pairs will also be suitable for imprinting analysis of other phylogenetically close strains commonly used as laboratory mice (e.g. 129/SvJ).

For imprinting studies, the use of reciprocal crosses between genetically distant mouse strains is of particular utility, since it allows for allele-specific DNA methylation and gene expression analyses based on DNA sequence polymorphisms (22,46). Importantly, our method preserves allelic information for the most commonly used BL6 × CAST cross and, moreover, it does so with ultra-deep allelic coverage (∼20 000-fold was achieved). Allele-specific IMPLICON has now been optimized for 13 ICRs and future work will surely expand this set for the rest of ICRs in this hybrid cross. This could also be envisioned for other hybrid crosses (e.g. BL6 versus *Mus musculus molossinus* JF1) (59), commonly used in imprinted studies using our defined criteria for primer design (see Materials and Methods). With the ultra-deep allelic coverage achieved, we believe our method will be better at discerning subtle parental allele-specific methylation changes as a result of environmental perturbations, pathological conditions or ageing, which might never have been sufficiently appreciated using other less powerful imprinting assays (Supplementary Table S1).

Diagnostics in human medicine is undoubtedly an area where analysis of imprinting methylation is important. Besides the 8 syndromes currently characterized for which the affected imprinted loci have been identified, some patients have recently been shown to display multi-locus imprinting disturbances (MLIDs). MLIDs are characterized by epimutations in several imprinted loci and clinical manifestations of, at least, one imprinting disorder (reviewed in (11)). Screening for MLIDs, that might remain underdiagnosed, is an obvious application for our human IMPLICON method which currently covers most of the human imprinted regions and could be extended to all regions, including the *DLK1-DIO3* region in the near future. Moreover, our IMPLICON method provides an easy and quick diagnostic tool not only for MLIDs, but also for other human conditions where altered imprinting is expected to be implicated, namely fetal growth restriction or cancer.

Another instance where inspection of imprinting methylation is becoming fashionable is in stem cell biology. Indeed, genomic imprinting has been shown to gain distorted patterns through stem cell conditions and upon reprogramming of somatic cells into hiPSCs. This creates an epigenetic obstacle for their correct use in disease modelling and their application in regenerative medicine (38,50–52,54). In contrast to blood samples and primary dermal fibroblast, hESCs and hiPSCs exhibit several imprinting defects consistent with the reports in the literature (Supplementary Table S4) (55). This was exacerbated when hESCs were grown in naïve conditions, where loss of methylation in imprinted regions followed the globally reduced levels of DNA methylation typical of cells grown in these culture conditions (47,49). Our results show the ability of the IMPLICON method to detect these methylation deficiencies in human stem cells. Since epigenetic stability in stem cells remains an issue and genomic imprinting provides an excellent thermostat of epigenetic fidelity, IMPLICON emerges as a simple and fast method for routine screening of hESC/hiPSCs ahead of their use in downstream applications.

In summary, we present a rapid method to measure imprinting methylation in a high-throughput and cost-effective manner that surpasses the limitations of other high-throughput sequencing methods when imprinting inspection is at stake (Supplementary Table S1). With further developments, IMPLICON could easily cover all known ICRs. Importantly, the guidelines and rules presented are extensive to screen DNA methylation profiles in any other genomic regions where high coverage is desired. With unprecedented coverage and nucleotide resolution, IMPLICON could become a gold-standard method to profile imprinting methylation in laboratory and clinical settings where aberrant imprinting has been or is believed to be implicated.

## DATA AVAILABILITY

All the sequencing datasets produced in this study are available at Gene Expression Omnibus GSE146129 (https://www.ncbi.nlm.nih.gov/geo/query/acc.cgi?acc=GSE146129).

## Supplementary Material

gkaa567_Supplemental_Files

## Notes

Present address: Tajda Klobučar, National Institute of Chemistry, Ljubljana, Slovenia.

Present address: Ferdinand von Meyenn, Institute of Food, Nutrition and Health, ETH Zurich, 8603 Schwerzenbach, Switzerland.

Present address: Elisa Kreibich, European Molecular Biology Laboratory (EMBL) Heidelberg, Germany.

Present address: Duarte Pólvora-Brandão, Chronic Diseases Research Center (CEDOC), NOVA Medical School | Faculdade de Ciências Médicas, Universidade Nova de Lisboa, Rua do Instituto Bacteriológico 5, 1150-–190, Lisboa, Portugal.
