# Supplementary material for: IMPLICON: an ultra-deep sequencing method to uncover DNA methylation at imprinted regions
Source: Nucleic Acids Res. 2020 Jul 4;48(16):e92. doi: 10.1093/nar/gkaa567 (PMC7498334; doi:10.1093/nar/gkaa567)
Supplement: gkaa567_Supplemental_Files [file gkaa567_supplemental_files.zip › Klobucar_SUPPLEMENTARY_INFORMATION.pdf]

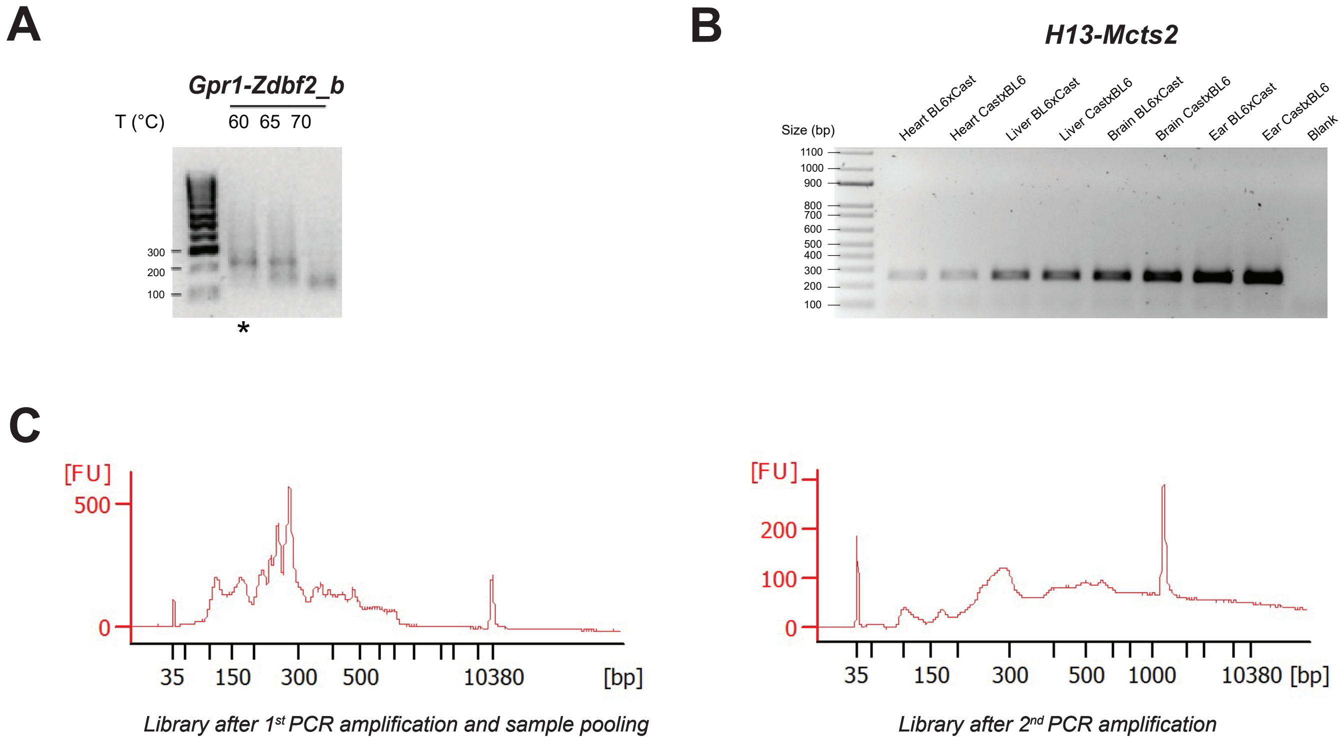

Supplementary Figure 1

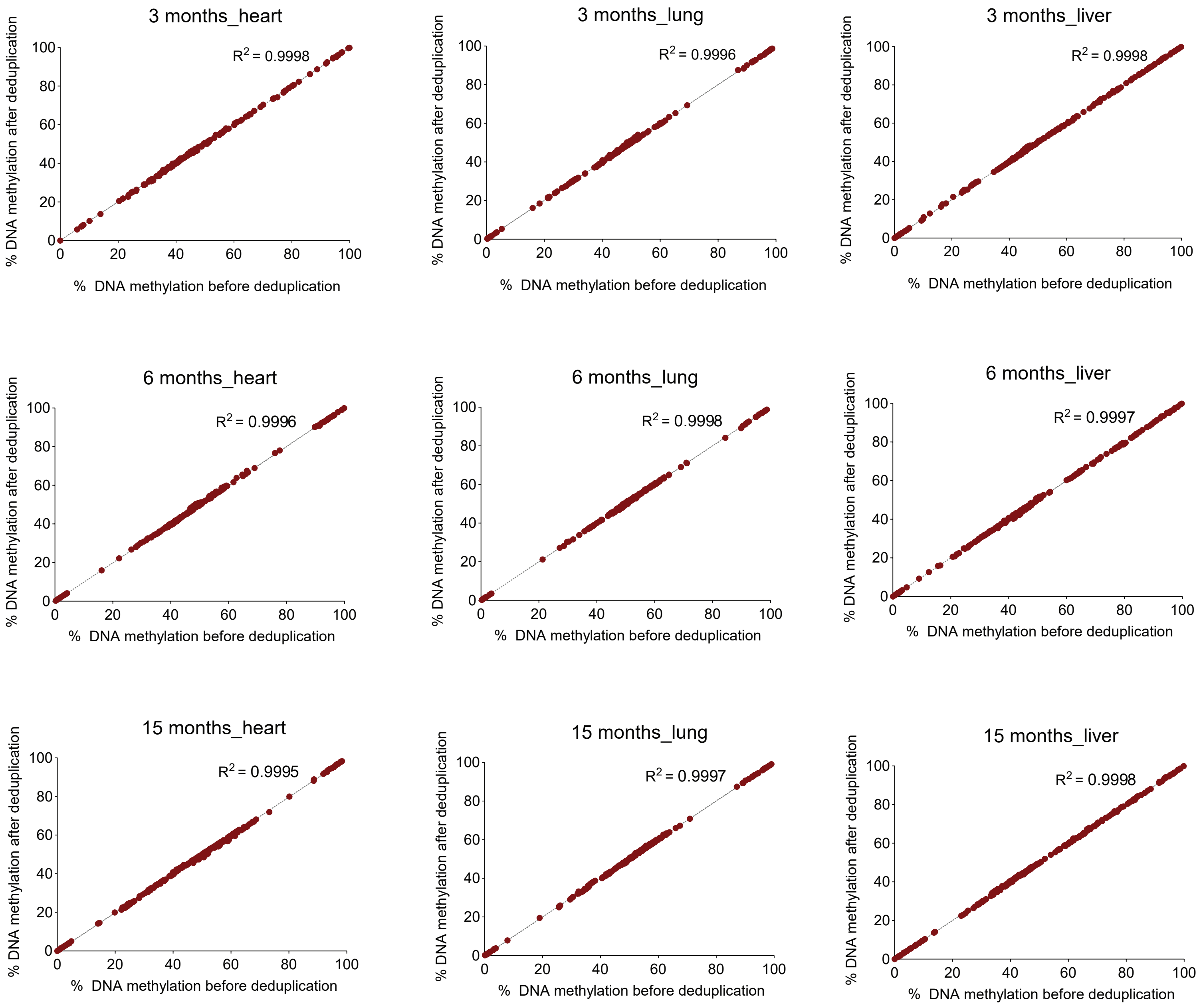

Supplementary Figure 2

Mouse_allelic-specific

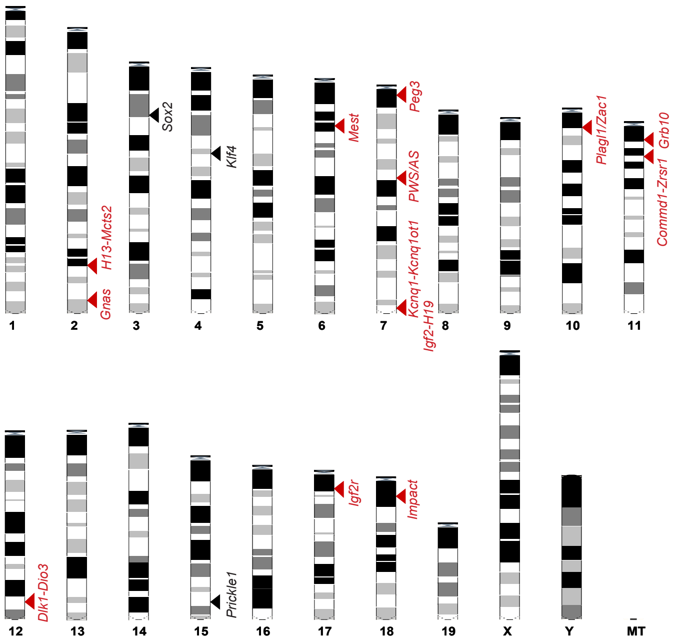

Supplementary Figure 3

Human

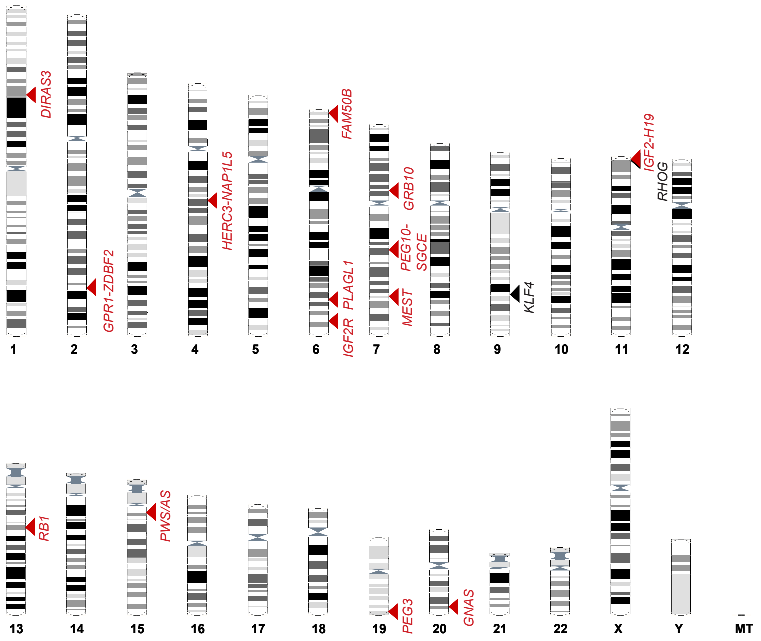

Supplementary Figure 4

SUPPLEMENTARY FIGURE LEGENGS

Suppl. Fig. 1 – Steps of quality control of the IMPLICON method.

A. Agarose gel displaying the primer optimization step for 1st PCR for the *Gpr1_Zdbf2_b* primer pair on *Gpr1-Zdbf2* locus; primer pair was tested with different annealing temperatures in mouse ESCs; *represents conditions chosen for the IMPLICON run.
B. Agarose gel displaying an example of individual PCR reactions after the 1st PCR step, amplifying *H13-Mcts2* locus in different tissues (heart, liver, brain, ear) from F1 hybrid mice; Blank represents a negative water control.
C. Library profiles obtained upon analysis on Agilent bioanalyzer after 1st PCR amplification and sample pooling (left) and after a 2nd PCR amplification and clean-up (right).

Suppl. Fig. 2 - Scatter plots showing overlapping percentages of DNA methylation values before and after a deduplication step in the analysis for inbred mouse libraries.

Suppl. Fig. 3 – Schematic view of the murine karyotype depicting the location of the regions detected by allele-specific IMPLICON; black arrowheads – control regions; red arrowheads – imprinted regions.

Suppl. Fig. 4 – Schematic view of the human karyotype depicting the location of the regions detected by the human version of IMPLICON; black arrowheads – control regions; red arrowheads – imprinted regions.

Technique	Throughput	Costs	Time	Bisulfite Conversion	Single CpG resolution	Advantages for imprinting analysis	Disadvantages for imprinting analysis	References
WGBS - Whole genome bisulfite sequencing or MethylC-seq	Low	Very High	2-4 weeks	Yes	Yes	Virtual representation of all imprinted regions at single base resolution	Low coverage of imprinted regions (~15x); Elaborate bioinformatics	Xie et al., 2012
RRBS - reduced representation bisulfite sequencing	Low	High	2-4 weeks	Yes	Yes	Greater coverage compared to WGBS at the imprinted regions represented at single base resolution	Absence of few imprinted clusters due to low genome coverage (10%); Elaborate bioinformatics	Stelzer et al., 2013
MeDIP-seq - Methylated DNA Immunoprecipitation sequencing	Low	High	2-4 weeks	No	No	No 5hmC detection	Low base resolution; biases towards hypermethylated regions	Proudhon et al., 2012
Illumina Infinium MethylationEPIC array	Medium	Medium	1-2 weeks	Yes	No	Catalog of MethylationEPIC probes for fast screening of human imprinted regions	Relative measurement; High signal to noise ratio	Hernandez Mora et al., 2018
Long-read Nanopore Sequencing	Low	Very High	2-4 weeks	No	Yes	Long reads; Direct 5mC detection	Low coverage of imprinted regions (~10x); Elaborate bioinformatics	Gigante et al., 2019
LR-EM-seq – Long Range Enzymatic Methyl-seq	Low	High	~1 week	No	Yes	Long reads, 5mC and 5hmC detection	Adapted only to 4 imprinted regions so far	Sun et al., 2019 (BioRxiv)
IMPLICON	High	Low	< 1 week	Yes	Yes	Ultra-deep genomic coverage (>1000 reads) at single allele and single base pair resolution	A few imprinted regions to be added	This work

Suppl. Table 1 - Advantages and disadvantages of current high-throughput methods to quantify DNA methylation at imprinted regions.

Abbreviations: 5hmC – 5'hydroxylmethyl-cytosine; 5mC – 5'methyl-cytosine

Imprinted cluster	Disease associated	Nazor et al., (2012): Infinium 450K BeadChip	Ma et al., (2014): Infinium 450K BeadChip	This study: IMPLICON
DIRAS3	-	Tendency for Hypermethylation	Tendency for Hypermethylation	Normal
GPR1/ZDBF2	-	-	-	Normal
HERC3-NAP1L5	-	Tendency for Hypermethylation	Normal	Tendency for Hypomethylation
FAM50B	-	-	-	Tendency for Hypomethylation
PLAGL1	Transient Neonatal Diabetes Mellitus	Tendency for Hypomethylation	Tendency for Hypomethylation	Tendency for Hypomethylation
IGF2R	-	-	-	Polymorphic imprinting
GRB10	-	Tendency for Hypomethylation	Normal	Rare Hypomethylation
PEG10-SGCE	Silver-Russell syndrome	Normal	Normal	Normal
MEST	-	-	Normal	Normal
IGF2-H19	Silver-Russell syndrome; Beckwith-Weideman syndrome	Tendency for Hypermethylation	Normal	Tendency for Hypermethylation
RB1	-	-	-	Hypermethylation
PWS-AS	Prader-Willy syndrome; Angelman syndrome	Tendency for Hypomethylation	Normal	Normal
PEG3	-	Hypermethylation	Hypermethylation	Hypermethylation
GNAS	Sporadic pseudohypoparathyreoidism Ib	Tendency for Hypomethylation	Rare Hypomethylation	Hypomethylation

Suppl. Table 4 - Comparative analysis of methylation defects at imprinted regions in human induced pluripotent stem cells from three methylome studies.
